# The Characteristics of Tremor Motion Help Identify Parkinson's Disease and Multiple System Atrophy

**DOI:** 10.3389/fneur.2020.00540

**Published:** 2020-07-10

**Authors:** Dongning Su, Shuo Yang, Wanli Hu, Dongxu Wang, Wenyi Kou, Zhu Liu, Xuemei Wang, Ying Wang, Huizi Ma, Yunpeng Sui, Junhong Zhou, Hua Pan, Tao Feng

**Affiliations:** ^1^Department of Neurology, Beijing Tiantan Hospital, Capital Medical University, Beijing, China; ^2^China National Clinical Research Center for Neurological Diseases, Beijing, China; ^3^Department of Hematology and Oncology, Jingxi Campus, Beijing ChaoYang Hospital, Capital Medical University, Beijing, China; ^4^Hinda and Arthur Marcus Institute for Aging Research, Hebrew SeniorLife, Roslindale, MA, United States; ^5^Hinda and Arthur Marcus Institute for Aging Research, Harvard Medical School, Boston, MA, United States

**Keywords:** parkinson's disease, multiple system atrophy, tremor, diagnosis, harmonics

## Abstract

**Background/Objectives:** Distinguishing between Parkinson's disease (PD) and multiple system atrophy (MSA) is challenging in the clinic because patients with these two conditions present with similar symptoms in motor dysfunction. Here, we aimed to determine whether tremor characteristics can serve as novel markers for distinguishing the two conditions.

**Methods:** Ninety-one subjects with clinically diagnosed PD and 93 subjects with MSA were included. Tremor of the limbs was measured in different conditions (such as resting, postural, and weight-holding) using electromyography (EMG) surface electrodes and accelerometers. The dominant frequency, tremor occurrence rate, and harmonic occurrence rate (HOR) of the tremor were then calculated.

**Results:** Our results demonstrated that the tremor dominant frequency in the upper limbs of the MSA group was significantly higher than that in the PD group across all resting (*F* = 5.717, *p* = 0.023), postural (*F* = 13.409, *p* < 0.001), and weight-holding conditions (*F* = 9.491, *p* < 0.001) and that it was not dependent on the patient's age or disease course. The tremor occurrence rate (75.6 vs. 14.9%, χ^2^ = 68.487, *p* < 0.001) and HOR (75.0 vs. 4.5%, χ^2^ = 46.619, *p* < 0.001) in the resting condition were significantly lower in the MSA group than in the PD group. The sensitivity of the harmonic for PD diagnosis was 75.0% and the specificity was relatively high, in some cases up to 95.5%. The PPV and NPV were 95.2 and 75.9%, respectively.

**Conclusion:** Our study confirmed that several tremor characteristics, including the dominant tremor frequency and the occurrence rate in different conditions, help detect PD and MSA. The presence of harmonics may serve as a novel marker to help distinguish PD from MSA with high sensitivity and specificity.

## Introduction

Parkinson's disease (PD) is a common neurodegenerative disease characterized by the progressive loss of dopaminergic neurons in the substantia nigra (SN) (1). Currently, the diagnosis of PD depends largely on clinical manifestations and the experience of clinicians (2), resulting in a compromised diagnosis accuracy. Adler et al. reported that, for example, only 26% of untreated or not clearly responsive PD cases have been diagnosed in patients with less than a 5-year disease duration (3).

Multiple system atrophy (MSA) is a disease that is most often misdiagnosed as PD since it has many similar motor symptoms as PD, such as tremor, rigidity, bradykinesia, and gait disturbances (4, 5). It is critical to diagnose these two diseases accurately, as people with MSA often respond poorly to levodopa (6) and progress with poor prognoses (7, 8). Several studies have explored strategies to better distinguish PD and MSA. Krismer showed that the Sniffin Sticks test identification subscore has a high specificity and moderate sensitivity in distinguishing PD from MSA (9). Yamamoto et al. also revealed that the mean duration of motor unit potentials (MUPs) recorded by sphincter EMG in the MSA group was significantly longer than that in the PD group, and the sensitivity and specificity were 67 and 54%, respectively (10). Treglia et al. determined the pooled sensitivity and specificity of MIBG scintigraphy to be 89 and 77%, respectively (11). Using 18-F-fluorodeoxyglucose positron emission tomography (FDG-PET) with quantitative voxel-based statistical image analysis, Brajkovic et al. claimed that the diagnostic accuracy of FDG-PET was 93% for IPD. Oh et al. demonstrated that the cutoff value for the posterior putamen/ventral putamen intersubregional ratio (defined as the ratio of the non-displaceable binding potential of one striatal subregion to that of another striatal subregion) was >0.65 and that the sensitivity and specificity for differentiating MSA from PD with 18F-FP-CIT PET were 90 and 45%, respectively (12). However, both MIBG scintigraphy and PET are expensive and not available in all countries. A novel marker which is easily accessible and has both great sensitivity and specificity in distinguishing PD from MSA is thus in high demand.

Recent neurophysiologic examinations have markedly contributed to the understanding of diseases with tremor, such as Parkinsonian disorders (13). Such methods, known as tremor analysis, are useful for detecting tremor frequency and monitoring tremor intensity, and they might serve as diagnostic tools for the detection of disorders with tremor. Tremor analysis has been widely used to distinguish between essential tremor (ET) and PD (14). However, the effects of tremor analysis on the detection of Parkinsonian-type disorders (i.e., PD and MSA) remain unknown. Therefore, the aim of this study was to explore different characteristics of tremor motion and feasibility to use them as markers in distinguishing PD and MSA. Specifically, we hypothesized that: (1) the dominant tremor frequency, the occurrence rate of tremor in different states, and the occurrence rate of harmonics would be significantly different between MSA and PD cases; and (2) these tremor characteristics could be used to distinguish PD and MSA.

## Methods

### Subjects

A total of 91 subjects with PD and 93 age-matched subjects with MSA from the Department of Movement Disorders at Beijing Tiantan Hospital (Beijing, China) were included in our study. All of them provided written informed consent as approved by the Beijing Tiantan Hospital Institutional Review Board. The criteria of both the PD and MSA groups were: (1) aged between 25 and 80 years old and (2) a confirmed diagnosis of PD or MSA. The diagnosis of PD was based on the 2015 MDS diagnosis criteria (2). The diagnosis of MSA as well as the diagnoses of MSA with predominant Parkinsonism (MSA-P) and/or predominant cerebellar ataxia (MSA-C) was based on the second consensus statement for the diagnosis of MSA (4).

The exclusion criteria were as follows: (1) a history of brain surgery, especially pallidotomy or electrode implantation for deep brain stimulation; (2) a history of neuromodulation therapy, such as transcranial direct current stimulation (tDCS) and transcranial magnetic stimulation (TMS); (3) impaired cognitive function or mental problems leading to a failure to complete all the procedures; and (4) metal implants, such as deep brain stimulation (DBS) or pacemakers.

### Tremor Analysis

A six-channel Nicolet EDX system (Nicolet company, US) with 4 pairs of EMG surface electrodes and 2 accelerometers was used in this study (sensitivity: 100 μV/D, scanning speed: 100 ms/D, EMG low-frequency filter: 10.0 Hz, EMG high-frequency filter: 10.0 kHz, accelerometer low-frequency filter: 0.5 Hz, accelerometer high-frequency filter: 30 Hz).

During the analysis of the upper limbs, recording electrodes were applied bilaterally to the bellies of the flexor carpi ulnaris and the extensor carpi ulnaris. Reference electrodes were placed on the corresponding distal tendons. Two accelerometers were fixed on the dorsal sides of both hands, 2 cm proximal to the third metacarpophalangeal joint. The data were recorded in three states: (1) at rest: patients sat in an armchair with their forearms resting on the armrests, leaving the hands free in the air to measure hand tremors; (2) in a posture: patients raised their arms in front of their body with their wrists parallel with the ground; and (3) holding 1000 g: patients held their arms in front of them while holding 1000 g sandbags. Each state was recorded for 30 s. During the analysis of the lower limbs, the recording electrodes were applied bilaterally to the bellies of the tibialis anterior and gastrocnemius. Reference electrodes were placed on the corresponding distal tendons. Two accelerometers were fixed on the dorsal sides of both feet, 4 cm proximal to the third metacarpophalangeal joint. The data were recorded under two states: (1) at rest: patients sat calmly in a chair with their feet flat on the ground and relaxed completely; and (2) in a posture: patients sat in a chair with their toes touching the ground and heels suspended. Each state was recorded for 30 s.

### Data Collection

**Figure 1:**
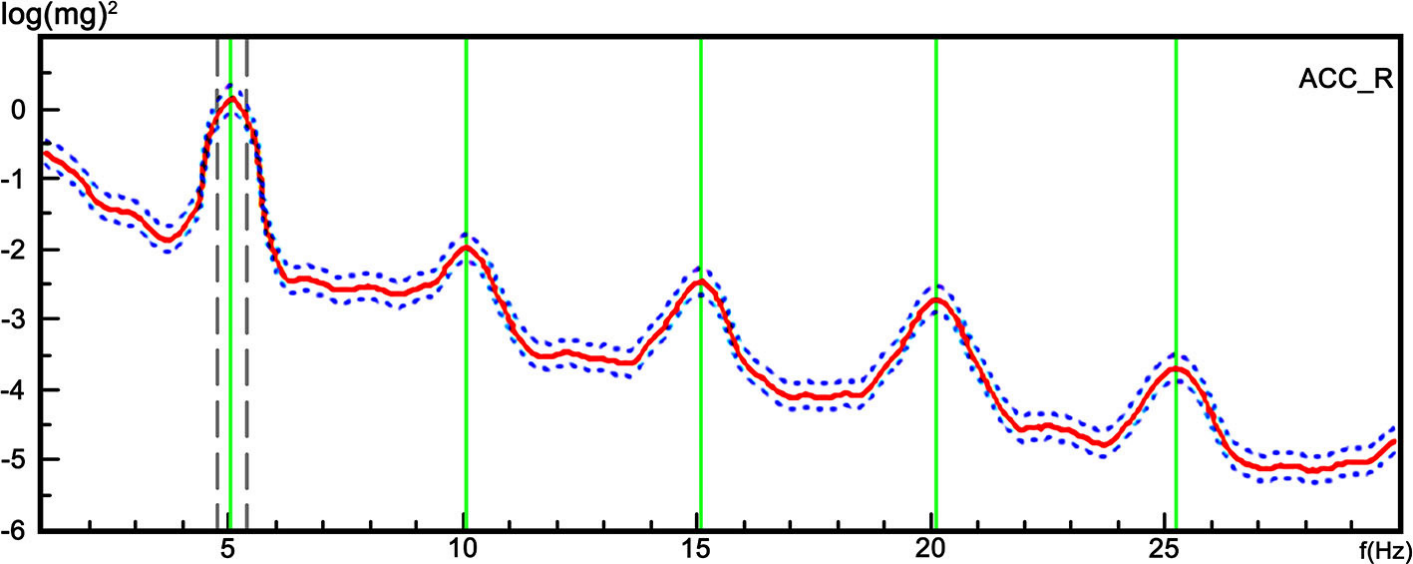
A representative trace of the tremor spectrum with harmonics from a single participant. The TRAS system converted the tremor data into a 1–30 Hz spectrum using Fourier transform. The X axis represents the frequency, and the Y axis represents the harmonics referring to the frequency contained in the spectrum, and was defined as the multiple integral of the fundamental wave.

The Tremor Recording and Analysis System (TRAS) (Nicolet company, US) was used to analyze the tremor signals. The system converted the tremor data into a 1–30 Hz spectrum by the Fourier transform (Figure 1). The frequency corresponding to the maximum peak was considered the dominant frequency. The harmonics corresponded to the frequencies contained in the spectrum, and was defined as the multiple integral of the fundamental wave. In upper limbs, the dominant frequency of the tremors and the harmonic occurrence rate were calculated under three conditions: at rest, in a posture, and holding 1000 g. For the lower limbs, the dominant frequency of the tremors and harmonic occurrence rate were recorded at rest and in the posture.

### Statistics

The measurement data are expressed as the mean ± standard deviation (*x* ± *s*). For every patient, the tremor scores and other tremor parameters were recorded on the more severe side. First, we used two sample *t*-tests to compare the demographics of the subjects between the PD and MSA groups, as well as between the MSA-P and MSA-C groups. Then, we used linear regression to compare the tremor frequency between the PD and MSA groups and excluded the influence of patient age and disease course. In addition, the MSA-P and MSA-C groups were compared in the same way. Finally, we used chi-square tests to compare the occurrence rates of resting tremors and harmonics between the PD and MSA groups, as well as between the MSA-P and MSA-C groups. SPSS 23.0 software was used to perform the analyses. *P* < 0.05 was regarded as statistically significant.

## Results

All subjects completed the tests. Within the MSA group, 65 subjects were diagnosed with MSA-P and 28 subjects were diagnosed with MSA-C. The subjects in the PD and MSA groups were sex- (χ^2^ = 0.196, *p* = 0.658) and age-matched (*F* = 1.470, *p* = 0.227). Although the disease courses were different between the PD and MSA groups (*F* = 71.996, *p* < 0.001), the UPDRS-III scores were similar (*F* = 0.687, *p* = 0.409). The demographics and clinical characteristics are shown in Table 1, and the scores of UPDRS-III tremor item are shown in Table 2.

**Table 1:** Clinical characteristics of the patients included in the study.

	**Subjects (Total)**	**Sex (male/female)**	**Age**	**Disease course**	**UPDRS-III**
PD	91	47/44	60.63 ± 10.04	8.16 ± 4.83	38.16 ± 18.84
MSA	93	45/48	60.49 ± 7.864	3.30 ± 1.479	30.2 ± 26.773

**Table 2:** UPDRS-III tremor scores in different states.

	**Resting tremor in upper limb**	**Posture tremor in upper limb**	**Loaded with 1000 g in upper limb**	**Resting tremor in lower limb**	**Posture tremor in lower limb**
PD	1.57 ± 1.52	2.03 ± 1.39	2.26 ± 1.41	1.04 ± 1.32	2.02 ± 1.30
MSA	0.52 ± 0.62	1.44 ± 0.79	2.04 ± 0.95	0.34 ± 0.68	1.69 ± 1.02
*P*-value	0.00	0.01	0.24	0.09	0.47

### Dominant Frequency

**Figure 2:**
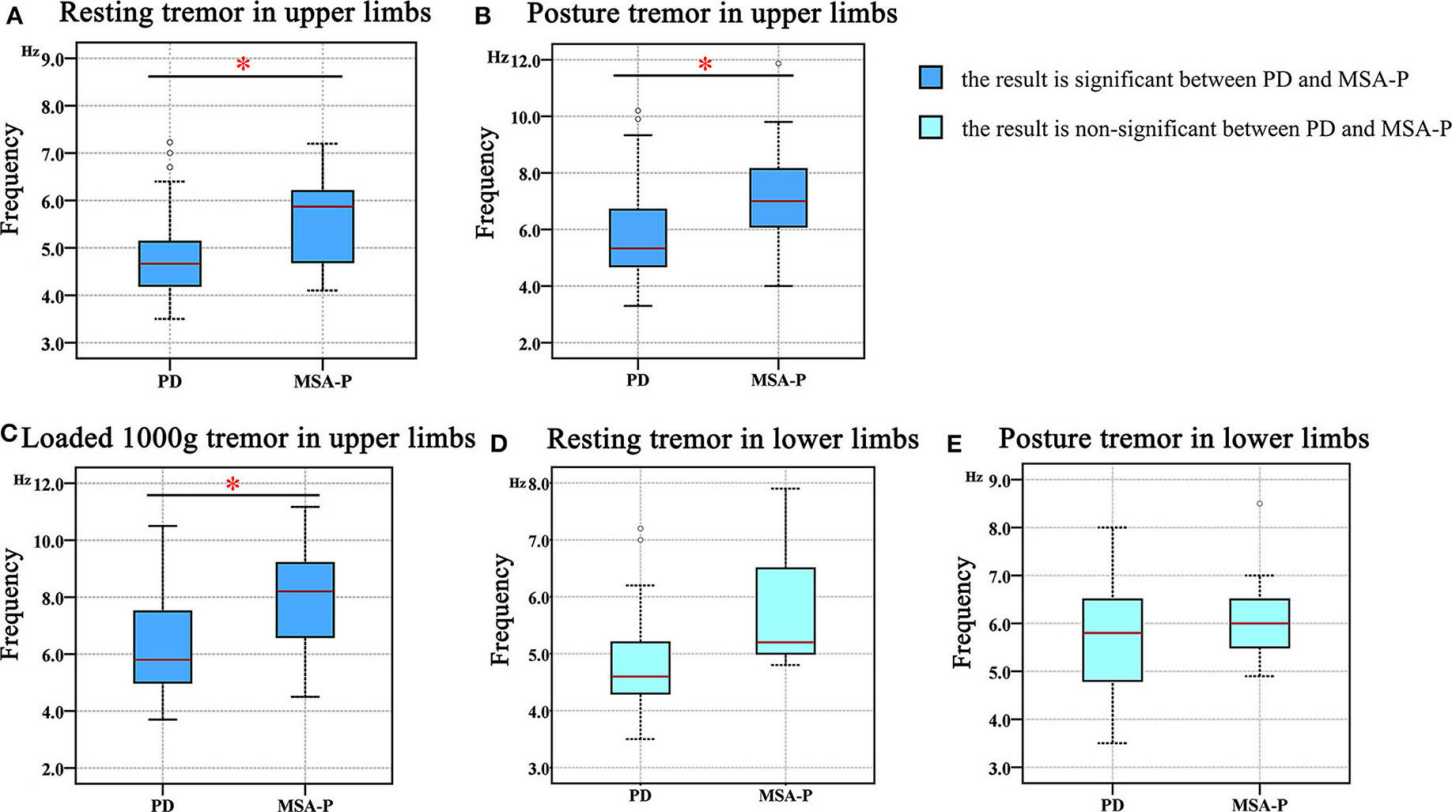
The difference in the tremor frequency between the PD and MSA groups. **(A)** Resting tremor in the upper limbs. **(B)** Postural tremor in the upper limbs. **(C)** Holding a load of 1000 g in the upper limbs. **(D)** Resting tremor in the lower limbs. **(E)** Postural tremor in the lower limbs. “*” in the figure represents a significant difference between the PD and MSA groups (*p* < 0.05).

Our results demonstrated that the tremor dominant frequency in the upper limbs of the MSA group was significantly higher than that in the PD group across all resting (*F* = 5.717, *p* = 0.023), posture (*F* = 13.409, *p* < 0.001), and weight-holding conditions (*F* = 9.491, *p* < 0.001) (Table 3, Figures 2A–C) and that it did not depend on the patient's age or disease course. However, this frequency in the lower limbs was not significantly different between the MSA and PD groups in either the resting (*F* = 0.314, *p* = 0.29) or posture state (*F* = 0.000, *p* = 0.74) (Table 3, Figures 2D,E).

**Table 3:** Dominant tremor frequency in different states.

	**Resting tremor in the upper limb**	**Postural tremor in the upper limb**	**Loaded with 1000 g in upper limb**	**Resting tremor in the lower limb**	**Postural tremor in the lower limb**
PD	4.76 ± 0.91	5.6 ± 1.36	6.22 ± 1.63	4.68 ± 0.82	5.58 ± 1.22
MSA	5.50 ± 0.95	7.27 ± 1.53	7.86 ± 1.75	4.49 ± 0.65	5.58 ± 1.10
*P*-value	0.02	0.00	0.00	0.29	0.74

**Figure 3:**
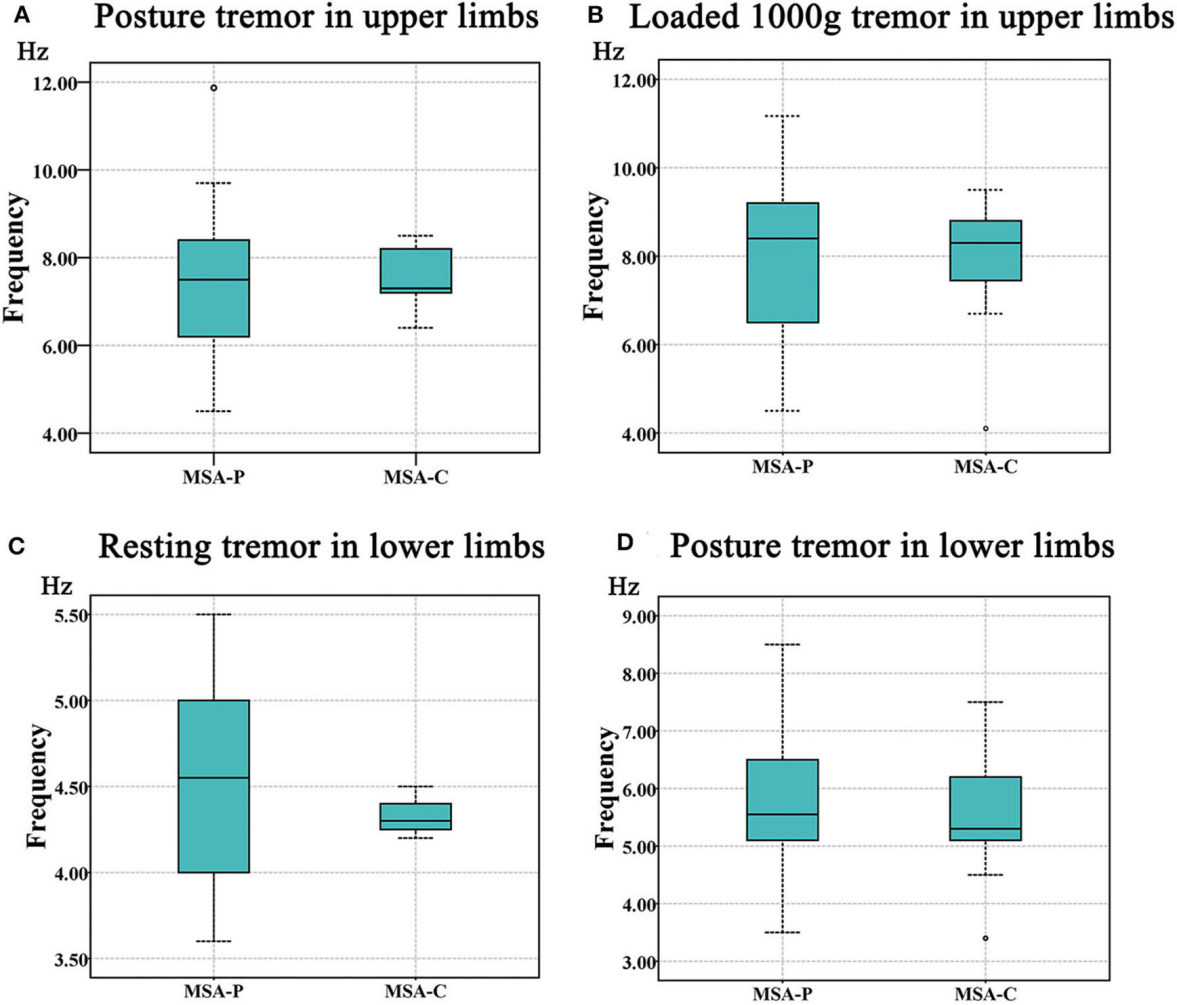
The comparison of tremor frequency between the MSA-P and MSA-C groups. **(A)** Postural tremor in the upper limbs. **(B)** Holding a load of 1000 g in the upper limbs. **(C)** Resting tremor in the lower limbs. **(D)** Postural tremor in the lower limbs. No significant difference was shown between the MSA-P and MSA-C groups (*p* < 0.05).

More specifically, we observed that the tremor dominant frequency in the upper limbs of the MSA-P group was not different from that in the MSA-C group in the posture (MSA-P: 7.24 ± 1.59 Hz, MSA-C: 7.52 ± 0.84 Hz; *p* = 0.711) and weight-holding conditions (MSA-P: 7.87 ± 1.75 Hz, MSA-C: 7.77 ± 1.86 Hz; *p* = 0.539) (Figures 3A,B). Since none of the MSA-C subjects presented typical resting tremors, the dominant frequency of resting tremors was not analyzed. Similar results were observed in the dominant frequency of resting and postural tremors in the lower limbs, that is, no significant differences were observed between the MSA-P and MSA-C subgroups. (MSA-P: 4.53 ± 0.70 Hz, MSA-C: 4.40 ± 0.28 Hz; *p* = 0.067 and MSA-P: 5.60 ± 1.14 Hz, MSA-C: 5.54 ± 1.05 Hz; *p* = 0.233) (Figures 3C,D).

### Occurrence Rate of Tremors in the Different States

The occurrence rate of resting tremors at a specific frequency in the PD group was significantly higher than that in the MSA group (75.6 vs. 14.9%, χ^2^ = 68.487, *p* < 0.001). The occurrence of resting tremors at a specific frequency was used to differentiate PD from MSA with a sensitivity of 75.6%, specificity of 85.1%, positive predictive value (PPV) of 82.9%, and negative predictive value (NPV) of 78.4%. However, the occurrence rates of postural tremors at a specific frequency were similar between the PD and MSA groups (86.7 vs. 71.3%, χ^2^ = 6.520, *p* = 0.686).

### Harmonics

**Figure 4:**
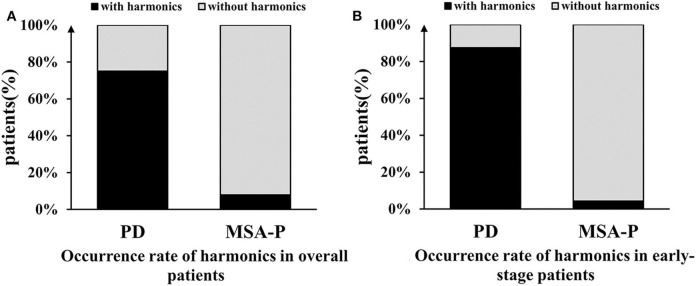
Comparison of the occurrence rate of harmonics between the PD and MSA groups. **(A)** The overall harmonic occurrence rate was 75.0% in the PD group, which was remarkably higher than that in the MSA-P group (4.5%). **(B)** In early-stage, the difference was more significant between the two groups.

The harmonic occurrence rates in the resting and postural states in the PD group were significantly higher than those in the MSA group: 71.1 vs. 4.5% for resting tremors (χ^2^ = 82.489, *p* < 0.001) and 65.8 vs. 3.0% for postural tremors (χ^2^ = 74.716, *p* < 0.001). No significant difference in the harmonic occurrence rates between the resting and postural states was found in the PD group (χ^2^ = 0.490, *p* = 0.484) or MSA group (χ^2^ = 0.214, *p* = 0.644). The overall harmonic occurrence rate, considering all the states together, was 75.0% in the PD group, which was remarkably higher than that in the MSA group (4.5%) (χ^2^ = 97.491, *p* < 0.001) (Figure 4). The sensitivity of harmonics for PD diagnosis was 75.0%, and the specificity was relatively high, in some cases up to 95.5%. The PPV and NPV were 95.2 and 75.9%, respectively. More details are presented in Table 4.

**Table 4:** Harmonics occurrence rate in the PD and MSP groups.

	**PD**	**MSA**	***p*-value**
Resting tremor	71.1%	4.5%	*p* < 0.001
Postural tremor	65.8%	3.0%	*p* < 0.001
Overall tremor	75.0%	4.5%	*p* < 0.001

No significant differences were found in the harmonic occurrence rates between the MSA-P subjects and MSA-C subjects (resting tremor: 7.9 vs. 0%, χ^2^ = 2.356, *p* = 0.257; posture tremor: 5.3 vs. 0%, χ^2^ = 1.537, *p* = 0.506; overall tremor: 7.9 vs. 0%, χ^2^ = 2.356, *p* = 0.257).

## Discussion

Our study confirmed that tremor characteristics, such as the dominant tremor frequency and tremor type (i.e., resting postural or action tremor), may provide meaningful information for diagnoses. We also provided, for the first time, evidence that harmonics can be helpful in distinguishing PD and MSA. Consistent with the previous results, our results confirmed that the tremor frequency in the MSA patients was significantly higher than that in the PD patients, regardless of which state the patients were in. Resting tremors occurred much less frequently in the MSA patients than in the PD patients. These markers show great promise in facilitating the differentiation of PD and MSA.

As previously reported, the most common tremor in patients with MSA is irregular, high-frequency postural tremor (15). The current study suggests that tremor frequency in PD and MSA groups is discrepant in the upper limbs but similar in the lower limbs. This highlights the potential for multiple oscillator involvement in tremor-genesis. Although this hypothesis has not been discussed carefully in MSA, PD studies using electromyographic recordings and accelerometry demonstrate results consistent with a multiple oscillator hypothesis (16, 17). The significant difference of tremor frequency in the upper limbs may help clinicians distinguish PD and MSA.

The occurrence rate of resting tremors in the MSA patients ranged from 12 to 44%, while in the PD patients, resting tremors were more frequent, with an occurrence rate of 65–88% (5, 18, 19). These differences in the neurophysiological characteristics of tremors in PD and MSA patients may result from differences in the pathologies of PD and MSA. *Song et al*. reported that the absence of resting tremors was related to severe atrophy of the globus pallidus (20). Helmich et al. believed that the globus pallidus played an important role in the onset of resting tremors in PD patients (21, 22), indicating that atrophy of the basal ganglia in MSA patients (23) might cause a low occurrence rate of resting tremors in MSA patients, which needs to be confirmed in future studies.

Multiple studies have explored using harmonics of tremor motion to identify different types of pathological tremors. Majchrzak et al. demonstrated that, compared to healthy controls, the harmonic index of tremor motions (the distance of the spectrum to the single narrow peak, which was normalized to the highest peak) was significantly higher in patients with Parkinsonian tremor, essential tremor, and cerebellar tremor (24). Jang et al. reported that the harmonic ratio (the value of the total harmonic peak power divided by the number of harmonic peaks) was remarkably lower in PD patients than in drug-induced Parkinsonism patients (25). In another study, *Muthuraman et al*. demonstrated that the average harmonic power can be used to distinguish advanced essential tremors from tremulous Parkinson's disease (26). Here, we show, for the first time, that the occurrence of harmonics is a marker that can be used to distinguish PD from MSA with a sensitivity of 75.0%, specificity of 95.5%, PPV of 95.2%, and NPV of 75.9%. Studies with larger sample sizes should be conducted in the future to confirm the results.

Research efforts have been made to explore the potential underlying mechanism that leads to harmonics in PT patients. For example, it is believed that the harmonics may arise from an asymmetry in the tremor waveforms (27). However, a neuroelectrical study using EEG and EMG showed there are differences in the topography of the cortical representations, that is, the corticomuscular delays in the dynamics of corticomuscular coherence, at both basic and harmonic frequencies. Therefore, both the basic and higher harmonic frequencies in Parkinsonian tremor patients may correspond to the oscillations arising from distinct central generators (28, 29). Studies need to be conducted in the future to further explore the underlying mechanism, which may also aid in the understanding of the reproduction of tremors in PD and MSA patients.

Although other measures, such as PET scans, could provide relatively high diagnostic accuracy for distinguishing PD and MSA, as mentioned in the introduction, these tests are expensive and not accessible for most patients. On the contrary, the tremor analysis with EMG and accelerometry is much cheaper and more convenient than functional imaging tests. Therefore, in our research, the role of tremor characteristics in distinguishing PD from MSA was further developed. Although the difference of tremor frequency in PD and MSA groups has been reported, the range of frequency could still overlap in these two diseases. Our study suggested that with the help of harmonics, clinicians could use tremor characteristics to distinguish PD from MSA accurately. The harmonics could be observed in TRAS system directly; consequently, it is a quick and easily accessible marker in clinical practice.

## Limitations

One limitation of our study is that none of the MSA-C patients had a specific resting tremor frequency. The range of the resting tremor frequency and resting tremor occurrence thus appeared to be 0% in the MSA-C group. Studies with larger sample sizes for the MSA-C cohort need to be conducted in the future to further explore the features of resting tremor in this group and confirm the findings reported in this study. Additionally, only the presence of harmonics (number of subjects) was used as a categorical variable in the analyses. The values of the harmonic index and harmonic ratio should be measured to determine whether such characteristics can aid in distinguishing PD from MSA. Besides, polymyoclonus mimic tremor, especially when it is rhythmic. To assess the presence of tremor, we observed the EMG result to make sure the involuntary motion was produced by antagonistic muscle contractions, and the frequency was relatively stable. Although our tremor analysis methods were consistent with the steps summarized in review, it is difficult to exclude the presence of polymyoclonus. Finally, the tremor characteristics could only help the diagnosis in patients with tremor. The differentiation between MSA and PD patients without tremor is of more clinical significance but is also more of a challenge. Future efforts are needed to explore this.

## Conclusion

In summary, our study confirmed that tremor characteristics, such as the dominant tremor frequency, and the occurrence rate in patients with different tremor types (i.e., resting postural or action tremor) provide helpful information for differentiating PD and MSA. Moreover, we found that the presence of harmonics may serve as a novel marker to help differentiate PD from MSA with high sensitivity and specificity.

## Data Availability Statement

All datasets generated for this study are included in the article/supplementary material.

## Ethics Statement

The studies involving human participants were reviewed and approved by Ethics Committee of Beijing Tiantan Hospital, Capital Medical University. The patients/participants provided their written informed consent to participate in this study.

## Author Contributions

DS, SY, HP, and TF contributed to the conception and design of the study. DS, SY, DW, WK, ZL, XW, YW, and HM collected the data. The statistical analysis and article draft were completed by DS. JZ, YS, and XW critically revised the article. HP and TF subsequently revised the article and approved the final manuscript. All authors contributed to the article and approved the submitted version.

## Conflict of Interest

The authors declare that the research was conducted in the absence of any commercial or financial relationships that could be construed as a potential conflict of interest.

## Abbreviations

PD: Parkinson's disease
MSA: multiple system atrophy
SN: substantia nigra
tDCS: transcranial direct current stimulation
TMS: transcranial magnetic stimulation
MSA-P: multiple system atrophy with predominant Parkinsonism
MSA-C: multiple system atrophy with predominant cerebellar ataxia
PPV: positive predictive value
NPV: negative predictive value.

## References

[1] Dickson DW. Neuropathology of Parkinson disease. Parkinsonism Relat Disord. (2018) 46(Suppl. 1):S30–3. 10.1016/j.parkreldis.2017.07.033 28780180 PMC5718208

[2] Postuma RB Berg D Stern M Poewe W Olanow CW Oertel W. MDS clinical diagnostic criteria for Parkinson's disease. Mov Disord. (2015) 30:1591–601. 10.1002/mds.26424 26474316

[3] Adler CH Beach TG Hentz JG Shill HA Caviness JN Driver-Dunckley E. Low clinical diagnostic accuracy of early vs advanced Parkinson disease: clinicopathologic study. Neurology. (2014) 83:406–12. 10.1212/WNL.0000000000000641 24975862 PMC4132570

[4] Gilman S Wenning GK Low PA Brooks DJ Mathias CJ Trojanowski JQ. Second consensus statement on the diagnosis of multiple system atrophy. Neurology. (2008) 71:670–6. 10.1212/01.wnl.0000324625.00404.15 18725592 PMC2676993

[5] Köllensperger M Geser F Ndayisaba JP Boesch S Seppi K Ostergaard K. Presentation, diagnosis, and management of multiple system atrophy in Europe: final analysis of the European multiple system atrophy registry. Mov Disorder. (2010) 25:2604–12. 10.1002/mds.23192 20922810

[6] Giagkou N Stamelou M. Therapeutic management of the overlapping syndromes of atypical parkinsonism. CNS Drugs. (2018) 32:827–37. 10.1007/s40263-018-0551-3 30051337

[7] Xie T Kang UJ Kuo SH Poulopoulos M Greene P Fahn S. Comparison of clinical features in pathologically confirmed PSP and MSA patients followed at a tertiary center. Parkinson's Dis. (2015) 1:15007. 10.1038/npjparkd.2015.7 28725681 PMC5516563

[8] Glasmacher SA Leigh PN Saha RA. Predictors of survival in progressive supranuclear palsy and multiple system atrophy: a systematic review and meta-analysis. J Neurol Neurosurg Psychiatry. (2017) 88:402–11. 10.1136/jnnp-2016-314956 28250027

[9] Krismer F Pinter B Mueller C Mahlknecht P Nocker M Reiter E. Sniffing the diagnosis: olfactory testing in neurodegenerative parkinsonism. Parkinsonism Relat Disord. (2017) 35:36–41. 10.1016/j.parkreldis.2016.11.010 27890451

[10] Yamamoto T Asahina M Yamanaka Y Uchiyama T Hirano S Fuse M. The utility of post-void residual volume versus sphincter electromyography to distinguish between multiple system atrophy and parkinson's disease. PLoS ONE. (2017) 12:e0169405. 10.1371/journal.pone.0169405 28060892 PMC5217958

[11] Treglia G Cason E. Meta-analysis on MIBG scintigraphy in differential diagnosis between Parkinson's disease and neurodegenerative parkinsonism. Parkinsonism Relat Disord. (2012) 18:805. 10.1016/j.parkreldis.2012.04.017 22575232

[12] Oh M Kim JS Kim JY Shin KH Park SH Kim HO. Subregional patterns of preferential striatal dopamine transporter loss differ in Parkinson disease, progressive supranuclear palsy, and multiple-system atrophy. J Nucl Med. (2012) 53:399–406. 10.2967/jnumed.111.095224 22323779

[13] Burne JA Hayes MW Fung VSC Yiannikas C Boljevac D. The contribution of tremor studies to diagnosis of Parkinsonian and essential tremor: a statistical evaluation. J Clin Neurosci. (2002) 9:237–42. 10.1054/jocn.2001.1017 12093126

[14] Bove F Di Lazzaro G Mulas D Cocciolillo F Di Giuda D Bentivoglio AR. A role for accelerometry in the differential diagnosis of tremor syndromes. Funct Neurol. (2018) 33:45. 10.11138/FNeur/2018.33.1.045 29633696 PMC5901940

[15] Levin J Kurz A Arzberger T Giese A Höglinger GU. The differential diagnosis and treatment of atypical parkinsonism. Dtsch Arztebl Int. (2016) 113:61–9. 10.3238/arztebl.2016.0061 26900156 PMC4782269

[16] Raethjen J Lindemann M Schmaljohann H Wenzelburger R Pfister G Deuschl G. Multiple oscillators are causing parkinsonian and essential tremor. Mov Disorders. (2000) 15:84–94. 10.1002/1531-8257(200001)15:1<84::AID-MDS1014>3.0.CO;2-K 10634246

[17] Scanlon BK Levin BE Nation DA Katzen HL Guevara-Salcedo A Singer C. An accelerometry-based study of lower and upper limb tremor in Parkinson's disease. J Clin Neurosci. (2013) 20:827–30. 10.1016/j.jocn.2012.06.015 23639618

[18] Kaindlstorfer C Granata R Wenning GK. Tremor in multiple system atrophy–a review. Tremor Other Hyperkinet Mov. (2013) 3. 10.7916/D8NV9GZ9 24116345 PMC3779823

[19] Wenning GK Geser F Krismer F Seppi K Duerr S Boesch S. The natural history of multiple system atrophy: a prospective European cohort study. Lancet Neurol. (2013) 12:264–74. 10.1016/S1474-4422(12)70327-7 23391524 PMC3581815

[20] Song YJC Huang Y Halliday GM. Clinical correlates of similar pathologies in parkinsonian syndromes. Mov Disorders. (2011) 26:499–506. 10.1002/mds.23336 21259341

[21] Helmich RC Janssen MJ Oyen WJ Bloem BR Toni I. Pallidal dysfunction drives a cerebellothalamic circuit into Parkinson tremor. Ann Neurol. (2011) 69:269–81. 10.1002/ana.22361 21387372

[22] Helmich RC. The cerebral basis of Parkinsonian tremor: a network perspective. Mov Disord. (2018) 33:219–31. 10.1002/mds.27224 29119634

[23] Krismer F Wenning GK. Multiple system atrophy: insights into a rare and debilitating movement disorder. Nat Rev Neurol. (2017) 13:232–43. 10.1038/nrneurol.2017.26 28303913

[24] Machowska-Majchrzak A Pierzchała K Pietraszek S Łabuz-Roszak B Bartman W. The usefulness of accelerometric registration with assessment of tremor parameters and their symmetry in differential diagnosis of parkinsonian, essential and cerebellar tremor. Neurol Neurochir Pol. (2012) 46:145–56. 10.5114/ninp.2012.28257 22581596

[25] Jang W Han J Park J Kim JS Cho JW Koh SB. Waveform analysis of tremor may help to differentiate Parkinson's disease from drug-induced parkinsonism. Physiol Meas. (2013) 34:N15–24. 10.1088/0967-3334/34/3/N15 23442947

[26] Muthuraman M Hossen A Heute U Deuschl G Raethjen J. A new diagnostic test to distinguish tremulous Parkinson's disease from advanced essential tremor. Mov Disord. (2011) 26:1548–52. 10.1002/mds.23672 21520285

[27] Deuschl G Lauk M Timmer J. Tremor classification and tremor time series analysis. Chaos. (1995) 5:48–51. 10.1063/1.166084 12780154

[28] Volkmann J Joliot M Mogilner A Ioannides AA Lado F Fazzini E. Central motor loop oscillations in parkinsonian resting tremor revealed magnetoencephalography. Neurology. (1996) 46:1359. 10.1212/WNL.46.5.1359 8628483

[29] Raethjen J Govindan RB Muthuraman M Kopper F Volkmann J Deuschl G. Cortical correlates of the basic and first harmonic frequency of Parkinsonian tremor. Clin Neurophysiol. (2009) 120:1866–72. 10.1016/j.clinph.2009.06.028 19748827

